# Paraneoplastic Pemphigus: A Paraneoplastic Autoimmune Multiorgan Syndrome or Autoimmune Multiorganopathy?

**DOI:** 10.1155/2012/207126

**Published:** 2012-12-19

**Authors:** Vikram K. Mahajan, Vikas Sharma, Pushpinder S. Chauhan, Karaninder S. Mehta, Anju Lath Sharma, C. Abhinav, Gayatri Khatri, Neel Prabha, Saurabh Sharma, Muninder Negi

**Affiliations:** ^1^Department of Dermatology, Venereology & Leprosy, Dr. R. P. Govt. Medical College, Kangra, Tanda, Himachal Pradesh 176001, India; ^2^Department of Pathology, Dr. R. P. Govt. Medical College, Kangra, Tanda, Himachal Pradesh 176001, India; ^3^Department of Radiotherapy & Oncology, Dr. R. P. Govt. Medical College, Kangra, Tanda, Himachal Pradesh 176001, India

## Abstract

Paraneoplastic pemphigus (PNP), a clinically and immunopathologically distinct mucocutaneous blistering dermatosis, is a severe form of autoimmune multiorgan syndrome generally associated with poor therapeutic outcome and high mortality. This IgG-mediated disease is initiated by an obvious or occult lymphoproliferative disorder in most cases. Clinically severe mucositis, and polymorphic blistering skin eruptions, and histologically acantholysis, keratinocyte necrosis and interface dermatitis are its hallmark features. A 58-year-old female presented with recurrent, severe, recalcitrant stomatitis and widespread erosions/blistering lesions of one-year duration. Treatment with repeated courses of systemic corticosteroids at a peripheral center would provide temporary relief. She also had fever, productive cough, odynophagia and poor oral intake, herpes zoster ophthalmicus, pain in the abdomen, and watery diarrhea. An array of investigations revealed chronic lymphocytic leukemia (CLL), mediastinal and para-aortic lymphadenopathy, bronchiolitis obliterans, and vertebral osteoporosis/fractures. With the diagnosis of CLL-associated PNP she was managed with dexamethasone-cyclophosphamide pulse (DCP) therapy for 3 cycles initially, followed by COP regimen (cyclophosphamide, vincristine, and prednisolone) for 5 cycles. Remission is being maintained with chlorambucil and prednisolone pulse therapy once in 3 weeks with complete resolution of skin lesions and adequate control of CLL.

## 1. Introduction

 Paraneoplastic pemphigus (PNP) is a mucocutaneous disease due to immunological effects of the tumor on resident immune system rather than by direct tumor infiltration or tissue damage caused by metastasis [1]. Although no age group or gender is exempt, the affected individuals in most instances are between 45 and 70 years of age and are males [2, 3]. This IgG-mediated disease is initiated by an obvious or occult lymphoproliferative disorder in most cases. Clinically severe mucositis and polymorphic blistering skin eruptions and histologically acantholysis, keratinocyte necrosis, and interface dermatitis are its hallmark features. Immunoprecipitation and immunoblot testing will detect autoantibodies directed against a complex of four polypeptides (mainly plakin family proteins and desmogleins) with different molecular weights: periplakin (210 and 190 kDa), desmoplakins-I and II (250 and 210 kDa), bullous pemphigoid antigen-1 (BPAG-I, 230 kDa), and envoplakin-I (210 kDa) [3, 4]. Pulmonary involvement as bronchiolitis obliterans is frequent, mostly irreversible and often fatal [5, 6]. The varied clinical and immunological presentations have led to revisions of the original criteria (Table 1) of Anhalt et al. [7] for diagnosis of PNP over the years. The simplified and most referred diagnostic criteria are proposed by Camisa et al. [8] and include three major criteria: (1) polymorphic mucocutaneous eruptions, (2) concurrent internal neoplasia, and (3) serum antibodies with specific immunoprecipitation pattern, and three minor criteria: (1) histologic evidence of acantholysis, (2) direct immunofluorescence (DIF) showing intercellular and basement membrane staining (for IgG and Compliment), and (3) indirect immunofluorescence (IIF) staining with rat bladder epithelium for circulating autoantibodies. Presence of 3 major or 2 major and 2 minor of these criteria is considered diagnostic. Response to treatment is generally poor with significant morbidity and mortality. 

The disease occurs worldwide and about 450 cases have been reported in the literature between 1990 and 2011 [6]. It is not frequently reported in India; a PubMed and IndMed search on September 10, 2012, revealed only one report of phaeochromocytoma-associated PNP by Dave et al. [9] in the perspective of anesthetic management of their patient. In this paper we describe another case of PNP with an idea to share our experience of managing this case. 

## 2. Case Report

**Figure 1:**
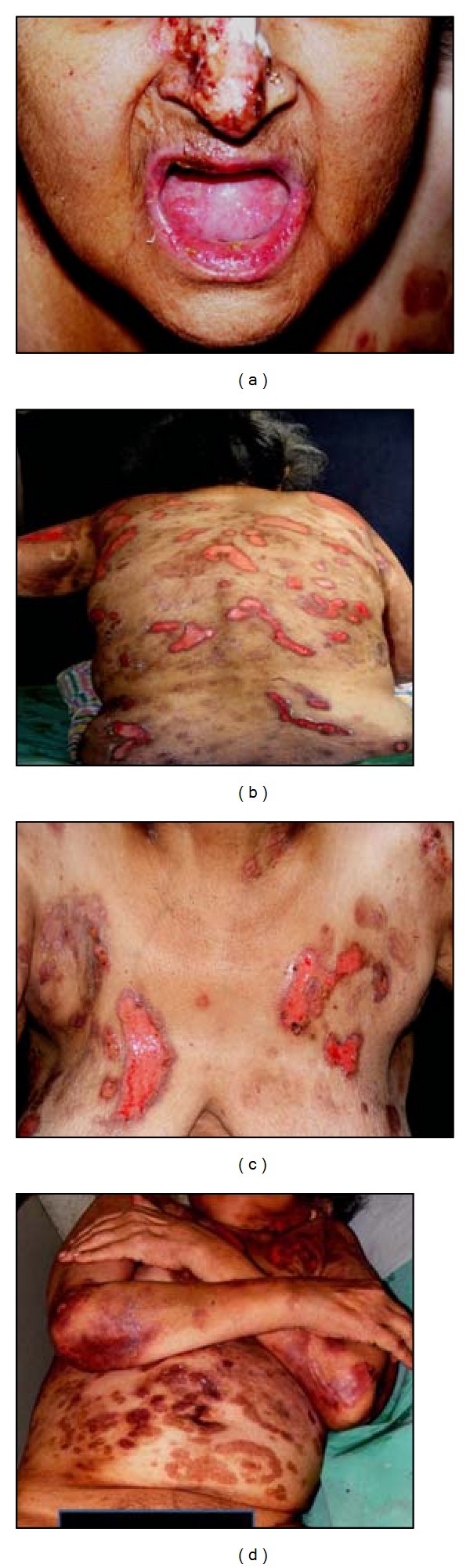
Severe erosions of vermilion lip and tongue, and nasal bridge (a), widespread crusted and denuded skin lesions over back (b), chest (c), abdomen and upper limbs (d).

**Figure 2:**
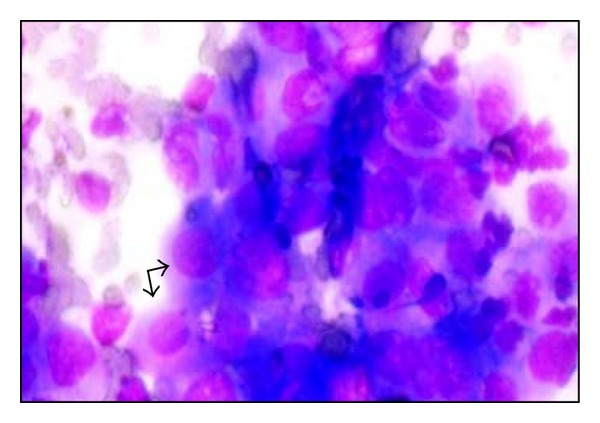
Acantholytic cells in Tzanck smear (*arrows*) (Giemsa, ×40).

**Figure 3:**
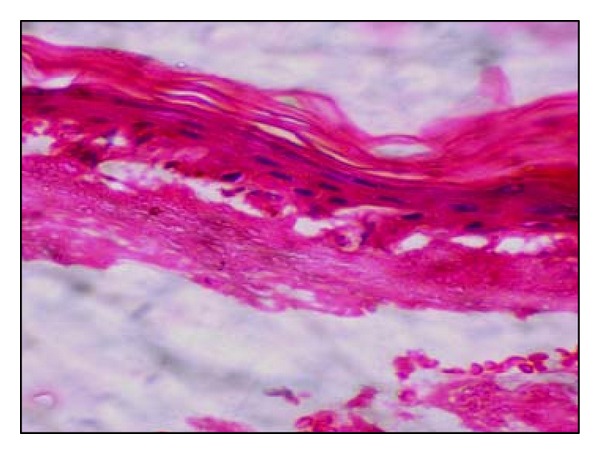
Histopathology of skin lesion shows mild spongiosis, edema, and predominantly subepidermal bulla and no inflammatory cell infiltrate (H&E, ×40).

**Figure 4:**
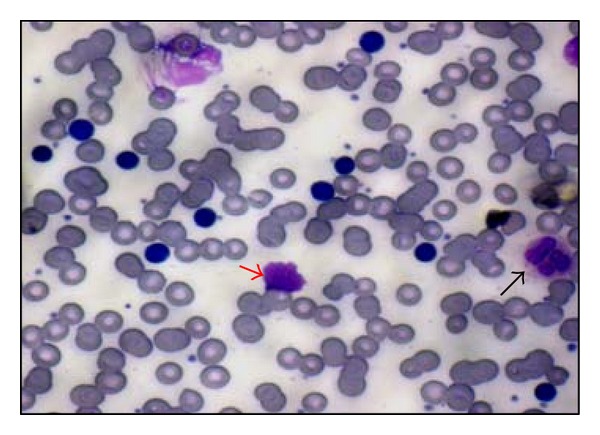
Peripheral blood film shows predominantly lymphocytosis, few smudge cells (*red arrow*), and occasional hypersegmented neutrophils (*black arrow*) suggestive of chronic lymphocytic leukemia.

**Figure 5:**
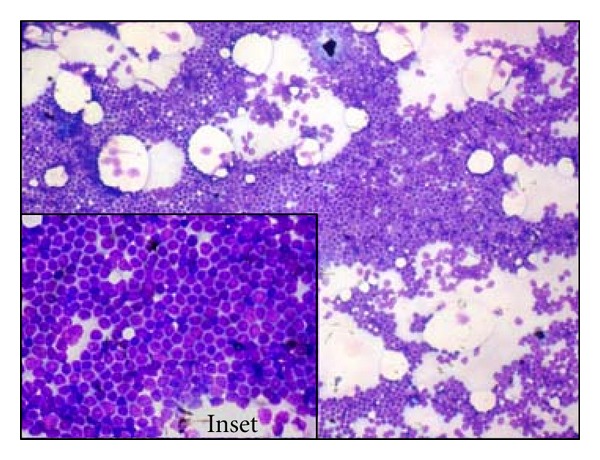
FNAC from axillary lymph mode showing monotonous population of small lymphoid cells admixed with occasional immunoblasts, mildly enlarged lymphoid cells with finely clumped chromatin and prominent nucleoli (Giemsa, ×10; *inset* ×40).

**Figure 6:**
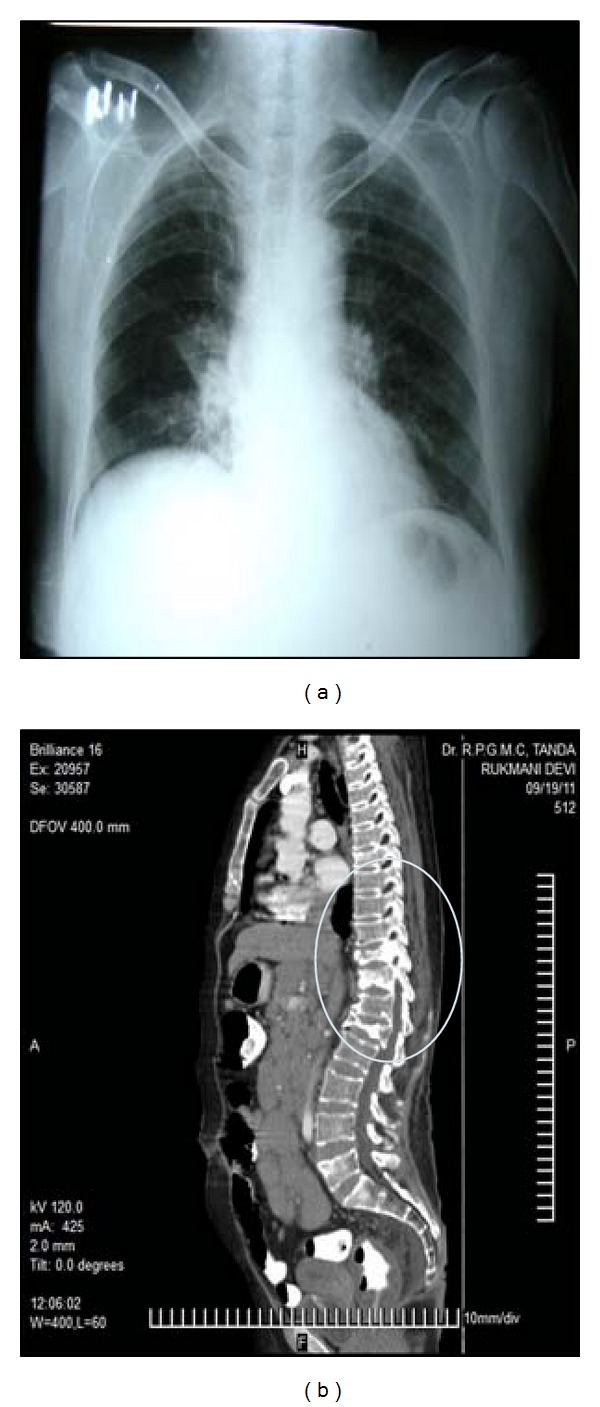
Segmental atelectasis in right parahilar region and bilateral hilar lymphadenopathy in X-ray chest (a). Vertebrae fractures of D10, D11, and L1 in CT scan (*encircled*) (b).

**Figure 7:**
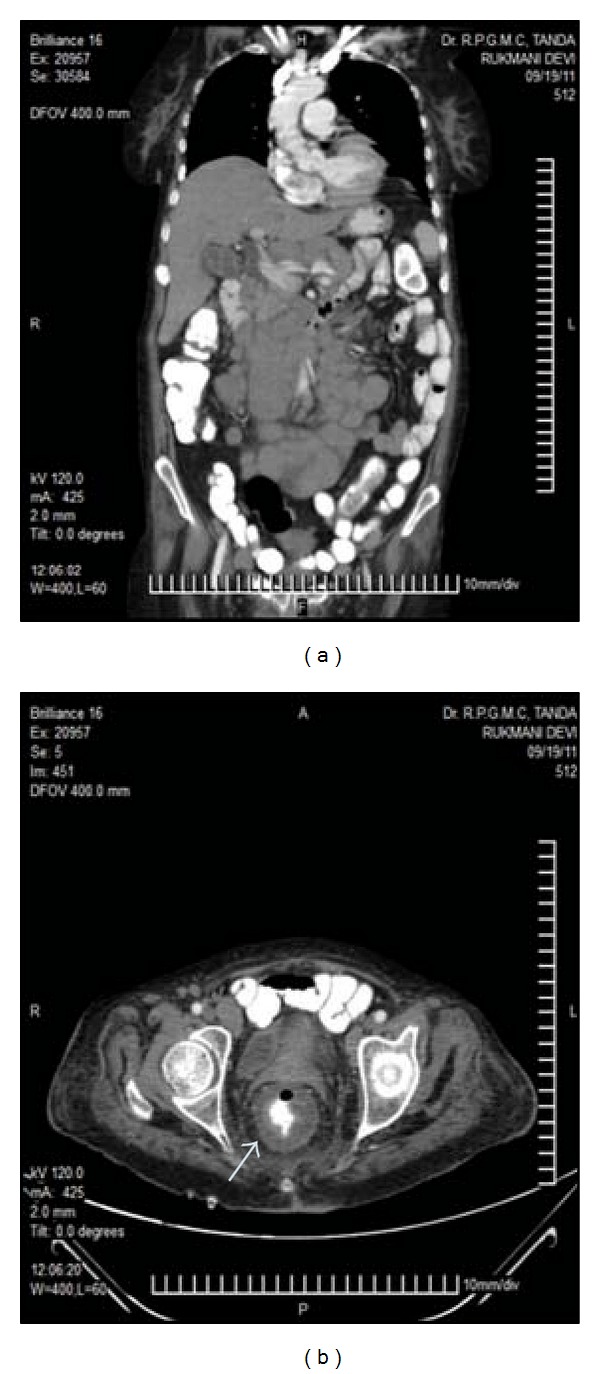
Retroperitoneal lymphadenopathy: multiple enlarged lymph nodes from epigastrium down to aortic bifurcation (a), and thickened sigmoidorectal wall (*arrow*) (b) in CT scan of abdomen.

 This 58-year-old female presented with recurrent, multiple, flaccid bullae, and erosions with oozing and crusting at places and severe painful oral ulcerations of 1-year duration. Various treatments including repeated courses of systemic corticosteroids at a peripheral center would provide temporary relief but recurrences and exacerbations were causing marked discomfort and disability. The cutaneous lesions were widespread and predominantly distributed over trunk and limbs. She had severe erosions/ulceration of the vermilion lips, palate and tongue (Figure 1), and conjunctival congestion (conjunctivitis). Oral lesions had preceded the skin lesion by 2 months. She also had episodes of moderate to high fever, productive cough and breathlessness, easy fatigability, bouts of abdominal pain, and watery diarrhea. She was sick, experienced pain while swallowing, dehydrated and had multiple crusted and denuded skin and few flaccid bullae over face, abdomen, back and limbs involving about 30% body surface area. The painful erosive lesions over right forehead were suggestive of herpes zoster ophthalmicus. Tzanck smear from a skin erosion and right forehead lesion showed acantholytic cells (Figure 2) and multinucleated giant cells (for herpes zoster), respectively. The deposition of IgG and complement in the basement membrane zone and intercellularly in the epidermis were seen on direct immunofluorescence (DIF) of perilesional skin. Histologic features of suprabasal acantholysis, dyskeratotic keratinocytes, and/or basal vacuolization and interface dermatitis (dyskeratotic keratinocytes, basal vacuolization, epidermal exocytosis) and absence of eosinophilic spongiosis and inflammatory infiltrate of eosinophils and neutrophils within the superficial dermis were suggestive of PNP (cf. pemphigus vulgaris). (Figure 3). Lab workup showed low hemoglobin (7.5 gm%), leukocytosis with predominant lymphocytosis (total leucocyte count 38900/cmm, L89%, N11%), elevated erythrocyte sedimentation rate (ESR 60 mm in 1st hr, Westergren method), and normal platelet counts (2,63000/cmm). Peripheral blood film showed dimorphic anemia and chronic lymphocytic leukemia (CLL) picture (Figure 4). Serum urea and creatinine were 59 mg% and 1.4 mg%, respectively. Hepatic functions, blood glucose, serum electrolytes, and urinalysis were essentially normal. Fine needle aspiration cytology from a small axillary lymph node showed features of small cell lymphocytic lymphoma/CLL (Figure 5). Radio-imaging studies (Figures 6 and 7) showed bilateral hilar lymphadenopathy and segmental atelectasis in chest X-rays and retroperitoneal lymph node enlargement involving those from epigastrium to aortic bifurcation and external iliac group, concentric thickening of sigmoido-rectal wall and fractured/collapsed vertebrae in ultrasonography (USG) and computed tomography (CT scan) of abdomen. The pathology of thickened sigmoido-rectal wall could not be ascertained as she did not consent for the colonoscopic biopsy due to ill health. 

### 2.1. Management and Course in the Hospital

With the diagnosis of CLL-associatedparaneoplastic pemphigus, bronchilitis obliterans, and iatrogenic (steroid induced) vertebral fractures, she was put on treatment with dexamethasone-cyclophosphamide-pulse (DCP) therapy (dexamethasone 100 mg in 500 mL of 5% dextrose given by a slow IV infusion over 2-3 hours on three consecutive days along with 500 mg of cyclophosphamide IV on the second day and repeat DCP doses at 3-week interval). Prednisone 40 mg/d and cyclophosphamide 50 mg/d PO during intervening days between two DCPs were added. She also received aciclovir 800 mg five times/d and amoxiclav 1 gm twice/d for 1 week, probiotics, nutritional supplements, and other supportive measures. For CLL, cyclophosphamide was considered adequate. During next 2 months of hospitalization most of her mucocutaneous lesions showed epithelization/healing, oral intake of food/fluids and general wellbeing improved, and she had no recurrence of diarrhea. After 3 DCPs she was put on COP regimen (single intravenous dose of cyclophosphamide 1 gm and oncovin/vincristine 1 mg, and prednisolone 20 mg, t.i.d × 7 d, PO). All her skin lesions had healed and new lesions stopped coming but she developed leucopenia (TLC 2400/cmm) after 7 COP doses. She received 8th COP dose after blood counts improved (TLC 7000/cmm) following transfusion of 2 units of whole blood and 3 doses of human granulocyte colony stimulating factor (G-CSF 300 *μ*m/d) administrated subcutaneously. Subsequently, she is being treated with chlorambucil (5 mg/d × 5 days) and prednisolone 20 mg t.i.d. × 7 days in a month, PO. Her skin lesions are in remission, the sigmoido-rectal thickening has reduced and all involved lymph nodes are reduced in size and number as seen in repeat CT scan abdomen. 

## 3. Discussion

 Anhalt et al. [7] in 1990, suggested the name paraneoplastic pemphigus for a pemphigus variant with underlying neoplasms wherein painful mucosal ulcerations and polymorphous skin lesions develop and is characterized by pathogenic autoantibodies directed against desmoplakin-I, BPAG-I, envoplakin-I, and periplakin. Its clinical presentation often mimics a drug reaction, erythema multiforme, Stevens-Johnson syndrome, or toxic epidermal necrolysis and wide variety of morphologic variants have been recognized: pemphigus-like, bullous pemphigoid-like, erythema multiforme-like, graft-versus-host disease-like, lichen planus-like and pemphigu vegetans-like [5, 10, 11]. Almost 2/3 of the cases arise in the context of a known (benign or malignant) neoplasia and hematologic neoplasms constitute about 84% of PNP cases while others may follow cytotoxic drug therapy [12–14]. Chronic lymphocytic leukemia (18.4%), non-Hodgkin's lymphoma (38.6%), Castleman's disease (18.4%), and benign thymoma (5.5%) were among the most common neoplasms in one series of 163 cases [12]. Nonhematologic malignancies constitute nearly 58% cases and also include carcinomas of epithelial origin (8.6%), sarcomas of mesenchymal origin (6.2%), and malignant melanoma (0.6%) [3]. Reticulum cell sarcoma, retroperitoneal spindle cell sarcoma, and Waldenstrom's macroglobulinemia are other common associated neoplastic disorders [12, 14]. Interestingly PNP may be an initial presentation of underlying neoplasia or present as a late consequence after complete resection of neoplasia [14, 15]. Chronic lymphocytic leukemia (a B-cell malignancy due to clonal proliferation of a CD5+ subpopulation), a common hematologic malignancy especially among elderly (above 50 years of age) in the Western world, has the most common association with PNP [3, 14]. On the other hand, PNP may be a presenting feature of occult Castleman's disease in children [3, 10, 16]. Reportedly, pulmonary involvement (bronchilitis obliterans) is more common with Castleman's disease-associated PNP and was noted in 26 of 28 cases by Nikolskaia et al. [17] and 7 of 10 cases by Wang et al. [18]. There is enough evidence to suggest that this results from antibody-mediated (both humoral and cell-mediated autoimmunities) acantholysis in bronchial epithelium due to the presence of desmoplakin in respiratory tract [4]. Cutaneous histology shows significant variability corresponding to the clinical presentation and age of the lesion [14]. While in DIF combination of IgG and complement deposition along the basement membrane zone and/or in the epidermal intercellular spaces is seen, staining of rodent bladder epithelium for polyclonal IgG (subclasses) and light chains is a feature on indirect immunofluorescence (IIF) [6, 14]. However, immunoprecipitation or immunoblotting is the standard diagnostic test but both are not readily available for wide use. 

 Majority of these cases have been treated as pemphigus vulgaris for want of clinical suspicion. Nevertheless, they require extensive investigative workup to exclude associated neoplasia. Although our patient had severe mucocutaneous erosions/ulceration particularly of oral cavity/vermilion lip and cutaneous bullous/ulcerative lesions, and histology and DIF features suggestive of classic PNP, she remained undiagnosed for want of clinical suspicion. She was managed at a peripheral center as a case of pemphigus vulgaris with systemic corticosteroids without significant benefit despite their side effects. Bronchilitis obliterans which is far more common in Castleman's disease-associated PNP and gastrointestinal symptoms were uncommon features in her. Together with conjunctivitis all were suggestive of multiorganopathy. 

 For the autoimmune phenomenon treatment with systemic corticosteroids in high doses is needed; addition of cyclosporine-A, DCP therapy, cyclophosphamide, mycophenolate mofetil, or azathioprine will curtail steroid intake and their side-effects. Intravenous immunoglobulin (IVIG), rituximab and alemtuzumab, plasmapheresis, and photopheresis are some other modalities of promising efficacy [3, 6]. The skin lesions respond better than mucosal (oral/bronchial) lesions which are highly refractory to treatment. Additionally, treatment of the underlying neoplasia is of paramount importance. For the treatment of CLL options include chemotherapy, chlorambucil, COP or CHOP regimen, and fludarabine, given alone or in combination. However, there is no recommended drug regimen that is consistently effective. We treated our patient with DCP therapy initially as it was considered effective for both autoimmune phenomenon and CLL and later with COP/CHOP regimens after the acute phase subsided. Adequate remission has been maintained with chlorambucil in combination with prednisolone. However, ultimate benefit cannot be comprehended at the moment as prolonged survival is uncommon and depends upon underlying neoplasia.

 Czernik et al. [6] suggested that term “paraneoplastic pemphigus” does not adequately address this multiorgan syndrome of varied signs and symptoms. Moreover pulmonary involvement is not a usual feature of pemphigus. Bronchilitis obliterans is a common feature seen in 30–40% of PNP cases [1], while the involvement of mucous membranes of esophagus, stomach, duodenum, and colon is infrequent in contrast to their involvement in pemphigus. This combined with reports of glomerulonephritis, neurological involvement, and ocular involvement (conjunctivitis, symblepharon, and corneal scarring) in PNP are further suggestive of this being a disease of multiorgan system [19, 20]. We tend to agree with Czernik et al. [6] that “paraneoplastic autoimmune multiorgan syndrome (PAMS)” is more inclusive nomenclature. However, we believe that the term “paraneoplastic autoimmune multiorganopathy” both clinically and pathogenetically will be more precise, unambiguously encompasses the heterogeneous manifestations of this distinct paraneoplastic process, and is perhaps more appropriate to use. Nevertheless, our viewpoints remain open for discussion. 

## Figures and Tables

**Table 1:** Paraneoplastic pemphigus: original diagnostic criteria of Anhalt et al. [7].

(1) Painful mucosal erosions and a polymorphous skin eruption in the context of an occult or known neoplasm generating a spectrum of histological features	
(2) Intraepidermal acantholysis, dyskeratosis/keratinocyte necrosis, and vacuolar interface changes in histopathology	
(3) Deposition of IgG and complement in intercellular epidermal and basement membrane zone seen on direct immunofluorescence	
(4) Detection of serum autoantibodies to stratified squamous epithelia, columnar, and transitional epithelia by indirect immunofluorescence	
(5) Serum immunoprecipitation of a characteristic complex of four proteins (250, 230, 210, and 190 kDa) from keratinocytes transitional epithelia by indirect immunofluorescence	
